# Adverse effects of cell-free and concentrated ascites reinfusion therapy for malignant ascites: a single-institute experience

**DOI:** 10.1186/s12885-022-09298-6

**Published:** 2022-03-14

**Authors:** Misato Tsubokura, Yuko Adegawa, Minoru Kojima, Ryuji Tanosaki, Ryuzaburo Ohtake, Yuki Kase, Nao Iwashita, Moemi Kasane, Saori Nakabayashi, Sayaka Takeuchi, Ken Kato, Narikazu Boku, Yukihide Kanemitsu, Takuji Okusaka, Hiroyuki Fujimoto, Kan Yonemori, Hiroto Ishiki, Kimihiko Kawamura, Eriko Satomi, Hiromichi Matsushita

**Affiliations:** 1 Department of Laboratory Medicine, National Cancer Center Hospital, 5-1-1, Tsukiji, Chuo-ku, Tokyo, 104-0045 Japan; 2 Department of Hematopoietic Stem Cell Transplantation, National Cancer Center Hospital, 5-1-1, Tsukiji, Chuo-ku, Tokyo, 104-0045 Japan; 3 Department of Blood Transfusion and Cellular Therapy, National Cancer Center Hospital, 5-1-1, Tsukiji, Chuo-ku, Tokyo, 104-0045 Japan; 4 Department of Gastrointestinal Medical Oncology, National Cancer Center Hospital, 5-1-1, Tsukiji, Chuo-ku, Tokyo, 104-0045 Japan; 5 Department of Colorectal Surgery, National Cancer Center Hospital, 5-1-1, Tsukiji, Chuo-ku, Tokyo, 104-0045 Japan; 6 Department of Hepatobiliary and Pancreatic Oncology, National Cancer Center Hospital, 5-1-1, Tsukiji, Chuo-ku, Tokyo, 104-0045 Japan; 7 Department of Urology, National Cancer Center Hospital, 5-1-1, Tsukiji, Chuo-ku, Tokyo, 104-0045 Japan; 8 Department of Medical Oncology, National Cancer Center Hospital, 5-1-1, Tsukiji, Chuo-ku, Tokyo, 104-0045 Japan; 9 Department of Palliative Medicine, National Cancer Center Hospital, 5-1-1, Tsukiji, Chuo-ku, Tokyo, 104-0045 Japan

**Keywords:** Cell-free and concentrated ascites reinfusion therapy (CART), Malignant ascites, Adverse effects (AEs), Fever, Reinfusion rate

## Abstract

**Background:**

Cell-free and concentrated ascites reinfusion therapy (CART) is a strategy for improving various intractable symptoms due to refractory ascites, including hypoalbuminemia. CART has recently been applied in the treatment of cancer patients. This study was performed to assess the safety of CART in a single cancer institute.

**Methods:**

We retrospectively reviewed 233 CART procedures that were performed for 132 cancer patients in our institute.

**Results:**

The median weight of ascites before and after concentration was 4,720 g and 490 g (median concentration rate, 10.0-fold), The median amounts of total protein and albumin were 64.0 g and 32.6 g (median recovery rates, 44.9% and 49.0%), respectively. Thirty-three adverse events (AEs) were observed in 22 (9.4%) of 233 procedures; 30 of these events occurred after reinfusion. The most common reinfusion-related AEs were fever (13 events) and chills (10 events). Univariate analyses revealed no significant relationships between the frequency of AEs and age, sex, appearance of ascites, weight of harvested and concentrated ascites, the ascites processing rate (filtration and concentration), weight of saline used for membrane cleaning, amount of calculated total protein for infusion, or prophylaxis against AEs; the reinfusion rate of ≥ 125 mL/h or ≥ 10.9 g/h of total protein affected the frequency of AEs, regardless of the prophylactic use of steroids.

**Conclusions:**

The observed AEs were mainly mild reactions after reinfusion, which were related to a reinfusion rate of volume ≥ 125 mL/h, a simple indicator in practice, or total protein ≥ 10.9 g/h. Although our study was retrospective in nature and undertaken in a single institute, this information may be helpful for the management of cancer patients with refractory malignant ascites using CART.

**Supplementary Information:**

The online version contains supplementary material available at 10.1186/s12885-022-09298-6.

## Background

Malignant ascites is a severe complication in the advanced cancer, including ovarian, stomach, breast, colon, lung, pancreas, and liver cancer. It is caused by peritoneal carcinomatosis that increases vascular permeability and lymphatic obstruction, and massive liver metastasis, which causes portal hypertension and hypoalbuminemia through the occlusion of hepatic vein, cirrhotic change and cachexia [1]. Ascites is classified into two categories, exudative and transudative ascites. Malignant ascites is usually exudative, but transudative ascites is also experienced when there is massive liver metastasis [2]. Once ascites is generated, the quality of life of cancer patients deteriorates through subjective symptoms such as abdominal fullness and pain, weight gain, appetite loss, nausea, constipation, leg edema and shortness of breath.

The most effective approach to treat ascites caused by cancers is tumor mass reduction by surgical resection and systemic chemotherapy. Because malignant ascites is commonly caused by tumors refractory to anti-cancer treatment, pharmacotherapy such as diuretics is not effective in many cases. Thus, its elimination is the most common pragmatic method to control the symptoms. In clinical practice, paracenteses, rather than peritoneovenous shunts, are preferred [3]. However, repeated paracenteses induce hypoproteinemia through the elimination of proteins involved in ascites, which may lead to a worsening of edema and ascites, as well as fatigue.

Cell-free and concentrated ascites reinfusion therapy (CART), which has been approved by the Japanese national health insurance system since 1981, is a strategy to improve hypoalbuminemia which positively contributes to the management of refractory ascites [1]. The processing of ascites in CART is performed using two types of filters; the first filter has a pore size of 0.2 μm, which is much smaller in diameter than bacteria and cells. This filter is utilized to completely remove unwanted components, such as tumor cells, from the ascites. The second filter is used to reduce the ascitic weight by eliminating the liquid component, resulting in the self ascites-derived fluid with concentrated proteins. The first report on CART in Japan was published in 1977 [4]. CART was initially applied in the treatment of refractory ascites in liver cirrhosis [5]. It has also been applied for malignant ascites [1].

CART itself is expected to improve ascites-associated symptoms (e.g. abdominal distension, general fatigue, dyspnea and loss of appetite) as palliative therapy, as well as renal dysfunction, while avoiding the use of an albumin preparation [1, 6–9]. CART combined with chemotherapy against various malignancies has been reported to be associated with a survival advantage in comparison to chemotherapy alone [10–12]. CART was reported to improve sinusoidal obstructive syndrome after hematopoietic stem cell transplantation [13]. However, CART is associated with adverse events (AEs), including shock, hypotension, chest pain, abdominal pain, dyspnea and hyperammonemia at puncture and drainage, and fever, chill, shivering, nausea, hypertension and headache at reinfusion [8].

In this study, we reviewed the cases of cancer patients with malignant ascites who underwent CART in our institute, with a focus on AEs, in order to report the safety of CART.

## Methods

### Patients

This cross-sectional study analyzed 233 CART procedures, which were performed in 132 patients at National Cancer Center Hospital (NCCH) from April 2015 to September 2019.

The exclusion criteria were as the follows: total bilirubin above 2.0 mg/dL, ascites containing a large amount of hemoglobin, uncontrollable bleeding tendency, gastrointestinal bleeding, bacterial peritonitis, hepatic encephalopathy, eGFR < 30 mL/min/1.73 m^2^, severe heart failure, sepsis, body temperature ≥ 38.0 °C, ileus, ovarian cancer and severe immunodeficiency. CART was applicable if the case did not meet the exclusion criteria. The indication for CART was decided by the attending physicians.

### Procedure of CART

We utilized a modified CART procedure, known as KM-CART, which employs an external pressure system using suction equipment and also utilizes filter membrane cleaning [9, 14]. Briefly, abdominal paracentesis was performed using a 14-G, 30-cm central venous catheter inserted into the abdominal cavity under ultrasonographic monitoring. Ascites was collected in a sterilized collection bag supplied with heparin (2500U for 3 L of ascites) by gravity flow at the rate of 1.5 to 2 L/h. The filtration and concentration of ascites were performed using AHF-MOW for filtration and AHF-UNH for concentration (Asahi Kasei Medical Co., Ltd., Tokyo, Japan), respectively.

The concentrated ascites was reinfused with or without the prophylactic administration of anti-allergic agents, including corticosteroids. The prophylaxis and reinfusion rates, which was recommended to be 100 to 150 mL/h in Asahi Kasei’s protocol, were determined by the policies of each attending physician and department.

### Data collection

The information related to the patient’s background and their clinical course, such as age/sex, diagnosis, adverse events (AEs) at the time points of puncture/drainage and after the reinfusion, prophylaxis against AEs and the reinfusion rate of the volume of concentrated ascites were collected from medical charts. We counted CART-related AEs that occurred by the following morning and were determined by each attending physician. The information related to the processing (filtration and concentration) of ascites is also collected, such as the appearance of ascites (bloody, chylous or serous), the weight and concentrations of protein/albumin before and after the processing, the time required for the processing, and frequency of washing filtration membrane during the processing. AEs were evaluated according to Common Terminology Criteria for Adverse Events (CTCAE) Version 5.0, except for a fever, which was defined as body temperature ≥ 38 °C and a change of ≥ 1 °C after reinfusion, according to febrile non-hemolytic transfusion reaction [15]. Ascites was classified according to the difference in the albumin concentration in the serum and ascites (serum-ascites albumin gradient; SAAG); namely, high SAAG (equivalent to ≥ 1.1 g/dL) indicates transudative ascites, whereas low SAAG (< 1.1 g/dL) indicates exudative ascites.

### Statistical analyses

All statistical analyses were performed in EZR, which is a modified version of R Commander designed to add frequently used statistical functions in biostatistics [16]. Differences between each group were compared using Fisher's exact test. The correlation of continuous variables between the two groups was tested using the Pearson product-moment correlation coefficient. Student’s *t*-test was used to compare continuous variables. To investigate the cut-off values of the volume and protein injection rate for adverse events, a receiver operating characteristics (ROC) curve of the volume and protein injection rate per hour and adverse events was used. All p-values were 2-sided, and p-values of < 0.05 were considered to indicate statistical significance.

The missing data were due to an insufficient description in the electronic medical record and were considered to be missing completely at random. In individual analyses, we performed available case analyses using the cases with no missing data.

Of note, there were two patients who had undergone CART so many times, we considered them to be a potential source of bias and examined whether or not the results would change by excluding their data.

## Results

**Fig. 1:**
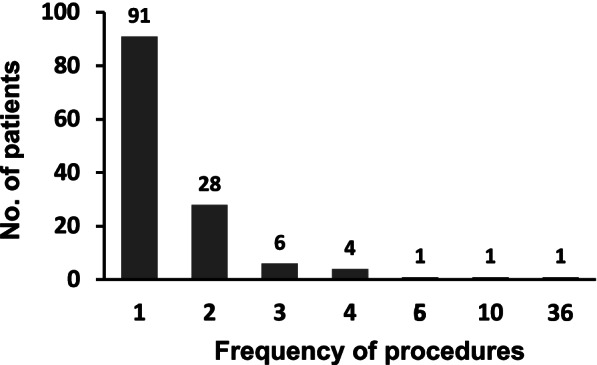
Frequency of CART procedures in each patient. The frequencies of CART in the 132 cases (233 procedures in total) are shown

The patients’ background characteristics are shown in Table 1. Notably, all patients suffered from malignant diseases. Among 132 patients, 41 (31%) received CART more than twice (Fig. 1). The median interval between repeated CART in these 41 patients was 21 days. Fifty-two of 132 patients (39%) had gastric cancer, accounting for 79 out of 233 procedures (34%). The numbers of patients with other malignancies were as follows: pancreatic cancer (n = 20; 15%) (22 procedures, 9%); colorectal cancer (*n* = 19; 14%) (66 procedures, 28%); biliary tract cancer (*n* = 5; 4%) (7 procedures, 3%); renal cancer patients (*n* = 4; 3%) (16 procedures, 7%); malignant melanoma (*n* = 4; 3%) (6 procedures, 3%); breast cancer (*n* = 3; 2%) (4 procedures, 2%); liposarcoma (*n* = 3; 2%) (4 procedures, 2%); malignant mesothelioma (*n* = 3; 2%) (3 procedures, 1%); small intestinal cancer (*n* = 3; 2%) (3 procedures, 1%); and appendiceal cancer (*n* = 2; 2%) (5 procedures, 2%).

**Table 1:** Patient characteristics

Characteritics		Number of patients	Number of CART procedures	
**total**		132	233	
**median age (range) (years)**		60 (27–82)		
**Sex**				
male	81			163
female	51			70
**background diseases**				
Gastric cancer	52			79
Pancreatic cancer	20			22
Colorectal cancer	19			66
Biliary tract cancer	5			7
Renal cancer	4			16
Malignant melanoma	4			6
Breast cancer	3			4
Liposarcoma	3			4
Malignant mesothelioma	3			3
Small intestinal cancer	3			3
Appendiceal cancer	2			5
Others	14			18

*CART* cell-free and concentrated ascites reinfusion therapy

**Fig. 2:**
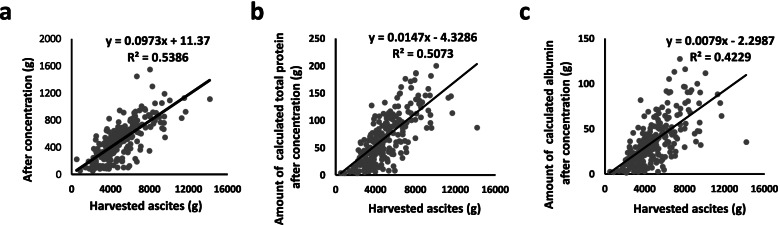
The recovery of the total protein/albumin content is correlated with the weight of harvested ascites. The relationships between the weight of harvested and concentrated ascites (**a**), between the weight of harvested ascites and the amount of calculated total protein after concentration (**b**), and between the weight of harvested ascites and the amount of calculated albumin after concentration (**c**) are shown. The approximation equation and correlation coefficient (R) are shown in each graph

The harvested ascites was classified as exudative (142 procedures, 61%) and transudative (91 procedures, 39%). The summary of ascites processing through filtration and concentration is shown in Table 2. The median weights of ascites before and after concentration were 4,720 (range, 500–14,150) g and 490 (range, 55–1,550) g, respectively. There was a correlation between these concentrations (*R*^*2*^ = 0.5386) (Fig. 2a), and the median concentration ratio was 10.0 (range, 2.2 to 55.0) times. The weights of harvested ascites tended to be correlated with the calculated amounts of total protein and albumin after concentration (*R*^*2*^ = 0.5073 and *R*^*2*^ = 0.4229, respectively) (Fig. 2b and c). The median concentrations of total protein and albumin in the concentrated ascites were up to 13.9 (range, 1.7–23.7) g/dL and 6.8 (range, 1.2–14.2) g/dL, respectively. Consequently, the calculated amounts of median total protein and albumin to infuse per procedure were 64.0 (range, 3.4–200.4) g and 32.6 (range, 1.5–127.7) g, and the recovery rates of total protein and albumin in the concentrated products were 44.9% (range, 9.8%-76.8%) and 49.0% (range, 11.6%-84.1%), respectively.

**Table 2:** Characteristics of concentrated ascites

					(*n* = 233)
Parameter		Harvested ascites	After concentration	Concentration ratio (times)	Recovery (%)
Total weight (g)	median	4,720	490	10.0	NA
	(min–max)	(500–14,150)	(55–1,550)	(2.2–55.0)	
Concentration of total protein (g/dL)	median	3.2	13.9	4.4	NA
	(min–max)	(0.3–6.3)	(1.7–23.7)	(0.6–31.0)	
Amount of calculated total protein (g)	median	152.4	64.0	NA	44.9
	(min–max)	(11.9–480.7)	(3.4–200.4)		(9.8–76.8)
Concentration of albumin (g/dL)	median	1.5	6.8	4.9	NA
	(min–max)	(0.1–3.4)	(1.2–14.2)	(0.7–38.0)	
Amount of calculated albumin (g)	median	69.0	32.6	NA	49.0
	(min–max)	(5.3–236.4)	(1.5–127.7)		(11.6–84.1)

*NA* not applicable

**Fig. 3:**
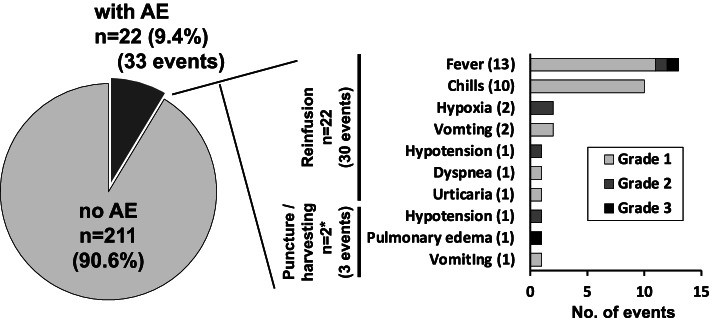
AEs of CART therapy. The left panel shows the frequency of procedures with AEs among the 233 procedures. The right panel shows the details of the AEs (33 events among 22 procedures) detected in this study. * AEs related to reinfusion also occurred in these 2 procedures

The AEs that were observed after reinfusion in this study are summarized in Fig. 3. Among 233 procedures, 33 events were detected in 22 procedures (9.4%). Regarding AEs related to reinfusion, 30 events occurred in 22 procedures (9.0%). Most reinfusion-related AEs were fever (13 events) and chills (10 events). Two events each of hypoxia and vomiting, and 1 event each of hypotension, dyspnea and urticaria were also experienced. Regarding AEs related to puncture/harvesting, 3 events occurred in 2 procedures (0.9%), where reinfusion-related AEs were also detected. These included pulmonary edema, hypotension and vomiting. There were two grade 3 events in all AEs, while all of the other events ranged from grade 1 to 2. All of these AEs, including the grade 3 events, were controllable either by conservative observation or transient medical treatment.

We then performed statistical analyses to clarify the factors related to these AEs (Table 3). Univariate analyses revealed that there was no statistically significant relationship between the frequency of AEs and age, sex, appearance (bloody, chylous or serous) and classification of ascites (exudative or transudative), weight of concentrated ascites, the processing ascites rate (filtration and concentration), the weight of saline used for cleaning the filter membrane (this part was analyzed in 225 procedures because the description in the medical record was missing for 8 procedures), and prophylaxis against AEs. A high weight of harvested ascites (≥ 4,721 g), and infusion of a large amounts of calculated total protein (≥ 64.1 g) and albumin (≥ 32.7 g) tended to be associated with an increased risk of experiencing AEs (*p* = 0.051, 0.051 and 0.075, respectively), although there was no statistical significance. Additionally, the reinfusion rate of volume of concentrated ascites (≥ 125 mL/h) and total protein (≥ 10.9 g/h) showed a statistically significant relationship with the occurrence of AEs in the analysis of 228 procedures (this part was analyzed in 228 procedures because the description in the medical record was missing for 5 procedures). It still showed statistical significance when the analysis was limited to infusion with the prophylactic use of steroids (equivalent to ≥ 100 mg hydrocortisone) (196 procedures because the records of the administration rate were missing for 4 procedures). Even after excluding the 46 procedures of the 2 patients who underwent CART 36 and 10 times from the re-analyses, the results were similar, with a statistical significance seen only in the reinfusion rate (Supplementary Table S1).

**Table 3:** The evaluation of the parameters associated with AEs in CART therapy

**paremeters**	**witn AEs**	**without AEs**	**p-value**
**Age (*n* = 233)**			
≤ 60 years old	12	110	1.000
≥ 61 years old	10	101	
**Sex (*n* = 233)**			
male	16	147	1.000
female	6	64	
**Appearance of havested ascites (*n* = 233)**			
bloody	4	41	0.390
chylous	0	20	
serous	18	150	
**Classificarion of havested ascites (*n* = 233)**			
exudative ascites	16	126	0.261
transudative ascites	6	85	
**Weight of havested ascites (*n* = 233)**			
≤ 4,720 g	7	110	0.051
≥ 4,721 g	15	101	
**Weight of concentrated ascites (*n* = 233)**			
≤ 490 g	8	110	0.183
≥ 491 g	14	101	
**Processing rate of ascites (*n* = 233)**			
≤ 89 mL/min	14	105	0.265
≥ 90 mL/min	8	106	
**Volume of saline used for washing filter membrane (*n* = 225)**			
≤ 450 mL	8	107	0.250
≥ 451 mL	13	97	
**Amount of calculated total protein for reinfusion (*n* = 233)**			
≤ 64.0 g	7	110	0.051
≥ 64.1 g	15	101	
**Amount of calculated albumin for reinfusion (*n* = 233)**			
≤ 32.6 g	7	111	0.075
≥ 32.7 g	15	100	
**Prophylaxis for AE (*n* = 233)**			
Steroids^a^	20	180	0.748
other than steroids^b^	2	31	
**Administration rate (*n* = 228)**			
≤ 100 mL/h	10	181	< 0.001
≥ 125 mL/h	12	25	
**Reinfusion speed of protein (*n* = 228)**			
≤ 10.8 g/h	3	109	< 0.001
≥ 10.9 g/h	19	97	
**Administration rate in cases treated with steroids**^a^ ***(n* = 196)**			
≤ 100 mL/h	10	156	< 0.001
≥ 125 mL/h	10	20	
**Reinfusion speed of proteinin cases treated with steroids**^a^ ***(n* = 196)**			
≤ 10.8 g/h	3	98	< 0.001
≥ 10.9 g/h	17	78	

*AE* adverse effects, *CART* cell-free and concentrated ascites reinfusion therapy

^a^equivalent to ≥ 100 mg hydrocortisone

^b^non-steroidal anti-inflammatory drugs, antihistamines, no prophylaxis, etc.

**Fig. 4:**
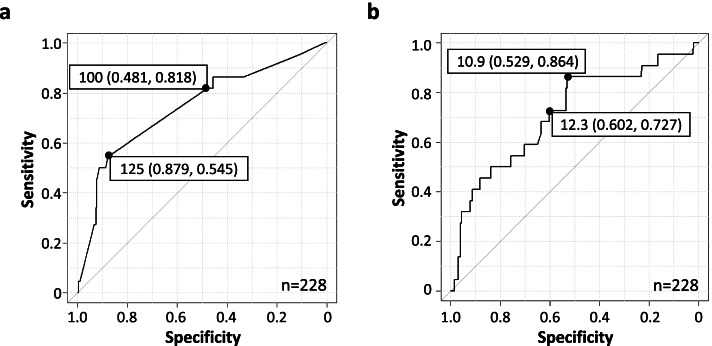
Results of an ROC analysis to determine the appropriate reinfusion rate without AEs. **a** The ROC analysis for the appropriate reinfusion rate of volume. The area under the ROC curve was 0.743. The sensitivity and specificity for 125 mL/h were 55% and 88%, respectively, and those for 100 mL/h were 82% and 48%, respectively. **b** The ROC analysis for the appropriate reinfusion rate of the protein dose. The area under the ROC curve was 0.713. The sensitivity and specificity for 12.3 g/h were 73% and 60%, respectively, and those for 10.9 g/h were 86% and 53%, respectively. The labels in the graphs include the cut-off value (specificity, sensitivity) without the units

To obtain the appropriate reinfusion rate of concentrated ascites, we analyzed the ROC curve-based sensitivity and specificity. When the cut-off value was defined as a point located with the shortest distance from the upper left corner on the ROC curve, the cut-off level was a volume of 125 mL/h, with a sensitivity and specificity of 55% and 88%, respectively. When the cut-off value was set at 100 mL/h, which was next to 125 mL/h in the list of reinfusion rates of volume with intermittent values and showed the highest value of sensitivity plus selectivity, the sensitivity was 82%, and the specificity was 48% (Fig. 4a). Regarding the reinfusion rate of total protein, the point located the shortest distance from the upper left corner on the ROC curve was 12.3 g/h, and the point with the highest value of sensitivity plus selectivity was 10.9 g/h. When 12.3 g/h was set as the cut-off value, the sensitivity and specificity were 73% and 60%, respectively, and when 10.9 g/h was used, the sensitivity and specificity were 86% and 53%, respectively (Fig. 4b).

**Fig. 5:**
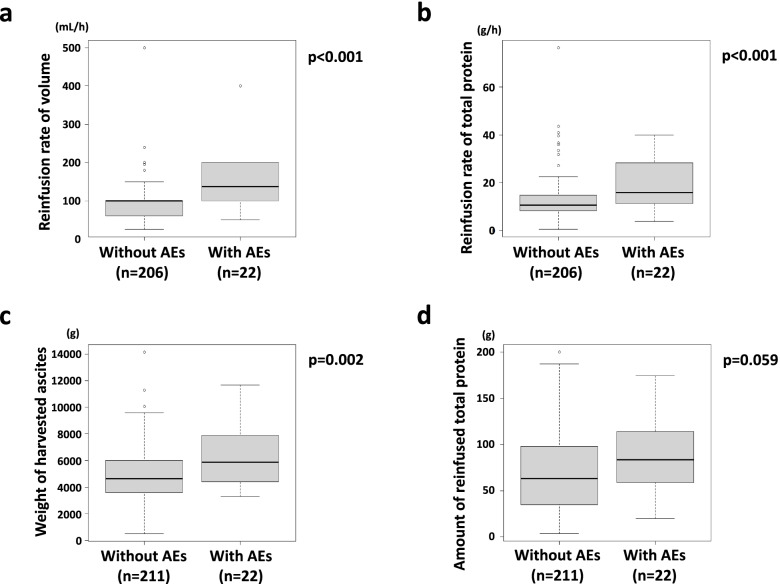
Differences in parameters of procedures with and without AEs. The reinfusion rate of volume (**a**) and total protein (**b**), weight of harvested ascites (**c**) and amount of calculated total protein for reinfusion (**d**) were compared between the procedures with and without AEs

Finally, we evaluated the reinfused rate of volume and total protein in the procedures with and without AEs. There were significant differences based on the presence of AEs (*p* < 0.001); the median value of the reinfused rate of volume was 138 mL/h (inter-quartile range [IQR] 100–180) with AEs versus 100 mL/h (IQR 60–100) without AEs, and the median value of reinfused rate of total protein was 15.9 g/h (IQR 11.2–24.3) with AEs and 10.7 g/h (IQR 8.2–14.7) without AEs, suggesting that the reinfused rate was closely related to the occurrence of AEs (Fig. 5a and b). The median value of the weight of harvested ascites was significantly greater with AEs (5865 g, IQR 4420–6710) than without AEs (4650 g, IQR 3660–6605) (Fig. 5c). In addition, the median value of the amount of reinfused total protein was greater with AEs (83.2 g, IQR 59.1–95.8) than without AEs (63.2 g, IQR 32.6–107.3), although not to a significant degree (*p* = 0.059) (Fig. 5d). These parameters, including the weight of harvested ascites and amount of reinfused total protein, were suspected to be related to the increase in the reinfused rate of total protein, although their effects might have been indirect.

## Discussion

One of the main purposes of CART in cancer treatment is to ease the discomfort derived from refractory ascites as a palliative treatment. Thus, the adverse effects related to CART, especially those with subjective symptoms, should be minimized in patients receiving the best supportive care. The current study showed that AEs were observed in 9.4% of 233 procedures. These were mainly mild and tolerable symptoms, like a fever and chills for grades 1 to 2. AEs were shown to corelate with the reinfusion rate of concentrated ascites. It is recommended that concentrated ascites be reinfused at a rate of ≤ 100 mL/h or ≤ 10.8 g/h of total protein, irrespective of prophylactic steroid administration.

AEs related to puncture/harvesting and reinfusion developed at the same frequency as seen in a previous multicenter study [12]. In the overall CART procedure, the most common AEs were fever and fever-related symptoms, such as chills after the reinfusion of concentrated ascites. The frequency of fever in this study, 5.6% (13 events), was comparable to that in previous studies, 5–16.4% [8, 11, 17, 18]. The average increase in body temperature was reported to be 0.1–0.44 °C [1, 9–11, 19]. Although a multi-center study showed that the risk of fever did not differ according to the conditions of reinfusion, such as the amount of reinfused ascites/protein or the speed of reinfusion [8], our study suggested that the reinfusion of larger amounts of protein may increase the risk of fever; this was similar to the finding of another report that analyzed patients with gynecologic cancer [17]. The multi-center study included cases of transudative ascites due to liver damage, such as liver cirrhosis and hepatic carcinoma. It is therefore hypothesized that fever may occur due to the enriched inflammatory cytokines in concentrated ascites derived from carcinomatous peritonitis. The ascitic concentration of inflammatory cytokines (e.g., IL-6, IL-8 and IL-10) did not have a significant correlation with the changes in body temperature [20]. Additionally, IL-6 in the ascites decreased in the process of filtration and concentration [21]. The exact causes of fever still require investigation.

Puncture/harvesting-related AEs were reported to be less frequent, occurring in 2.5% of procedures in the previous multi-center study, and 0.9% of the procedures in the present study (2 of 233 procedures). The AEs in our study included pulmonary edema, which might be caused by inflation of lungs due to elimination of a large amount of ascites, and hypotension, which might be caused by extravascular defluvium of the blood plasma component. The weight of harvested ascites in these 2 procedures was more than the median in this study, 6,665 mL and 11,535 mL, but improved with transient medical treatment. This could be life-threating, as it could injure vital organs and systems, and the severity might be dependent on the weight of harvested ascites, in addition to the general condition of each patient. Thus, appropriate management of patients, including careful observation and prompt treatment, are required when performing CART, especially when a large weight of ascites is harvested.

In order to avoid AEs, the manufacturer’s protocol recommends an ascites reinfusion rate of 100 to 150 mL/h. In most cases, reinfusion was performed after the prophylactic administration of corticosteroids for AEs, because steroids have been shown to be effective for inhibiting body temperature elevation in CART [8, 19]. However, AEs occasionally occurred, even with steroid prophylaxis. The finding in this study, that a reinfusion rate of 125 mL/h was the cut-off value from the ROC analysis and < 125 mL/h reduced AEs was consistently observed, regardless of the presence of steroid prophylaxis, suggesting the necessity for a stricter guideline regarding the reinfusion rate. For safety in the CART procedure, a reinfusion rate of ≤ 100 mL/h or ≤ 10.8 g/h for the protein dose, which ensured a sensitivity of ≥ 80%, was desired. CART has recently been applied for more cancer patients, who may have exudative ascites [1]. Although our study was retrospective in nature and performed in a single institute, this information may be helpful for the management of cancer patients.

One of the criticisms of our study was the low recovery rate of total protein and albumin in the processing of ascites (44.9 and 49.0%, respectively). In the modified CART (KM-CART) procedure, the recovery rates of albumin and globulin in 11 cancer patients were reported to be 71.1% ± 9.6% and 57.6% ± 7.1%, respectively, without membrane cleaning [14], while the protein recovery rate was 40.7% ± 14.0% in 4781 procedures with membrane cleaning [9], which were comparable to the data in this study. Frequent membrane cleaning therefore reduced the recovery rates of total protein and albumin. In addition, all patients in our study suffered from cancer. A multicenter study revealed that the lowest and middle tertile procedures of the recovery rate (47.3% ± 11.1%, 70.0% ± 6.0%) included a higher proportion of patients with exudative ascites (64.5%, 66.7%) and ascites with higher protein levels (3.6 ± 1.8 g/dL, 2.9 ± 1.4 g/dL), which was in sharp contrast to the highest tertile (89.8% ± 6.7%), included a lower proportion of patients with exudative ascites (32.4%) and demonstrated ascites with lower protein levels (2.2 ± 1.4 g/dL) [8]. The background of ascites in our study was similar to the lowest to middle tertiles rather than the highest tertile. Accordingly, our recovery rate was considered to be reasonable and realistic.

Another criticism was whether CART was performed safely, because more than two-thirds of the cases analyzed in this study actually received CART only once. The patients who were not eligible to receive active treatment in our institute were often followed at either a regional institute or a local clinic for palliative supportive care. Therefore, most patients only received CART a limited number of times at our institute.

A fever is a transient and mild AE in CART; however, its management is still important and valuable because CART is usually performed as a palliative therapy [1, 11]. Especially in cancer patients, the characteristics and contents of reinfused concentrated ascites vary according to those of the original ascites and modification through the CART procedure. It is therefore difficult to precisely perform a case–control study on CART for advanced cancer patients. Although the progress based on our findings might be limited, the accumulation of these findings will improve the strategy for CART, which is becoming increasingly important for relieving the discomfort associated with refractory ascites in cancer patients.

## Conclusion

In conclusion, the CART, a palliative therapy established to relieve ascites-associated symptoms by the re-use of collected ascites, was found to be a safe treatment strategy. Most AEs were mild and tolerable, being mainly evaluated as grades 1 to 2, and were related to a reinfusion rate of volume ≥ 125 mL/h, a simple indicator in practice, or total protein ≥ 10.9 g/h. Although this study was retrospective in nature and carried out at a single institute, this information may nevertheless be helpful in the management of cancer patients with refractory malignant ascites using CART.

## Supplementary Information

**Additional file 1: Table S1.** The evaluation of parameters associated with AEs in CART therapy excluding two cases who received CART more than 10 times.

## Data Availability

Data are available from the corresponding author upon reasonable request.

## Abbreviations

AE: Adverse events
CART: Cell-free and concentrated ascites reinfusion therapy
SAAG: Serum-ascites albumin gradient
